# Digenic Variants in the *TTN* and *TRAPPC11* Genes Co-segregating With a Limb-Girdle Muscular Dystrophy in a Han Chinese Family

**DOI:** 10.3389/fnins.2021.601757

**Published:** 2021-03-04

**Authors:** Qian Chen, Wen Zheng, Hongbo Xu, Yan Yang, Zhi Song, Lamei Yuan, Hao Deng

**Affiliations:** ^1^Center for Experimental Medicine, The Third Xiangya Hospital, Central South University, Changsha, China; ^2^Department of Pathology, The Third Xiangya Hospital, Central South University, Changsha, China; ^3^Department of Neurology, The Third Xiangya Hospital, Central South University, Changsha, China; ^4^Disease Genome Research Center, Central South University, Changsha, China

**Keywords:** limb-girdle muscular dystrophies, digenic variants, the *TTN* gene, the *TRAPPC11* gene, exome sequencing

## Abstract

Limb-girdle muscular dystrophies (LGMD) are hereditary genetic disorders characterized by progressive muscle impairment which predominantly include proximal muscle weaknesses in the pelvic and shoulder girdles. This article describes an attempt to identify genetic cause(s) for a LGMD pedigree via a combination of whole exome sequencing and Sanger sequencing. Digenic variants, the titin gene (*TTN*) c.19481T>G (p.Leu6494Arg) and the trafficking protein particle complex 11 gene (*TRAPPC11*) c.3092C>G (p.Pro1031Arg), co-segregated with the disease phenotype in the family, suggesting their possible pathogenicity.

## Introduction

Limb-girdle muscular dystrophies (LGMD) are hereditary genetic myopathies characterized by progressive muscle impairment, predominantly involving proximal muscle weakness in the pelvic and shoulder girdles (Narayanaswami et al., 2014; Angelini, 2020). LGMD’s variable clinical spectrum ranges from adult-onset weakness with slowly progressive muscle deterioration to severe infantile subtypes unable to walk unassisted. Cardiac and respiratory muscles may also be involved (Magri et al., 2017; Angelini, 2020). Disease prevalence is estimated up to 6.9/100,000 with a carrier frequency of up to 1/150 (Mahmood and Jiang, 2014; Winckler et al., 2019). Current diagnostic approaches rely primarily on a synthesis of clinical features, imaging characteristics, electrophysiological examination, biochemical data, muscle biopsy and genetic testing (Mahmood and Jiang, 2014; Iyadurai and Kissel, 2016). The level of serum creatine kinase (CK) varies from normal, through mildly elevated to highly elevated (>100× normal) (Iyadurai and Kissel, 2016; Liewluck and Milone, 2018). Classic pathological features of muscle biopsy show fiber caliber diversification, atrophy, hypertrophy, necrosis, phagocytosis, regeneration, and nuclear internalization (Cotta et al., 2014; Bastian et al., 2015; Liewluck and Milone, 2018). Muscle tissue loss and filling by fat and connective tissue were detected in advanced-stage patient muscle tissue (Cotta et al., 2014; Bastian et al., 2015).

As of this writing, at least 29 autosomal-inherited LGMD subtypes have been classified applying the updated definition. A new proposed LGMD classification of subtypes follows the formula: “LGMD, inheritance (R for recessive or D for dominant), order of discovery (number), affected protein” (Straub et al., 2018; Angelini, 2020).

LGMD2J, recently classified as LGMD R10 titin-related, caused by the titin gene (*TTN*) variants, is a serious autosomal recessive disease-causing proximal muscle weakness in childhood or adulthood. It deteriorates over the next 20 years ending in a wheelchair with high serum CK level (Evilä et al., 2014; Iyadurai and Kissel, 2016; Zheng et al., 2016a). LGMD2S, recently classified as LGMD R18 TRAPPC11-related, is caused by the trafficking protein particle complex 11 gene (*TRAPPC11*) variants. It is inherited in an autosomal recessive manner and presents as a gradually progressive proximal muscle weakness with childhood onset and high CK. Some sufferers, in addition to having the muscle weakness, present with intellectual disability, ataxia, ocular deficiency, and hepatic steatosis (Bögershausen et al., 2013; Nigro and Savarese, 2014; Fee et al., 2017; Wang et al., 2018).

This study describes a Han Chinese family with the digenic variants, *TTN* c.19481T>G (rs376857772, p.Leu6494Arg) and *TRAPPC11* c.3092C>G (rs200466260, p.Pro1031Arg) in the patients. Co-segregation analysis and bioinformatics prediction suggested that the digenic variants may be responsible for the LGMD phenotype.

## Materials and Methods

### Pedigree and Participants

**FIGURE 1:**
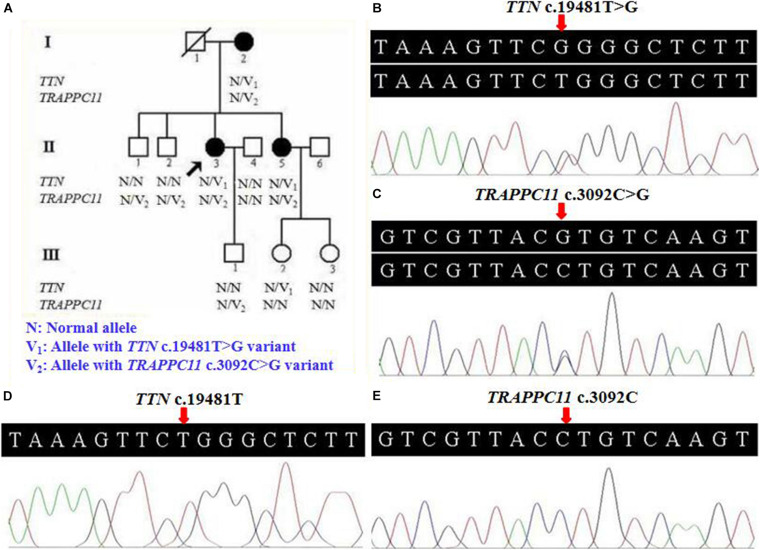
Pedigree and genetic data of the individuals in this study. **(A)** Pedigree of a Han Chinese three-generation family with LGMD. Arrow symbolizes proband; N, normal allele; V_1_, *TTN* c.19481T>G variant; V_2_, *TRAPPC11* c.3092C>G variant. **(B)** The sequencing diagram of heterozygous *TTN* c.19481T>G (p.Leu6494Arg) variant. **(C)** The sequencing diagram of heterozygous *TRAPPC11* c.3092C>G (p.Pro1031Arg) variant. **(D)** Sequence of normal control in the *TTN* gene. **(E)** Sequence of normal control in the *TRAPPC11* gene. LGMD, limb-girdle muscular dystrophies; *TTN*, the titin gene; *TRAPPC11*, the trafficking protein particle complex 11 gene.

**FIGURE 2:**
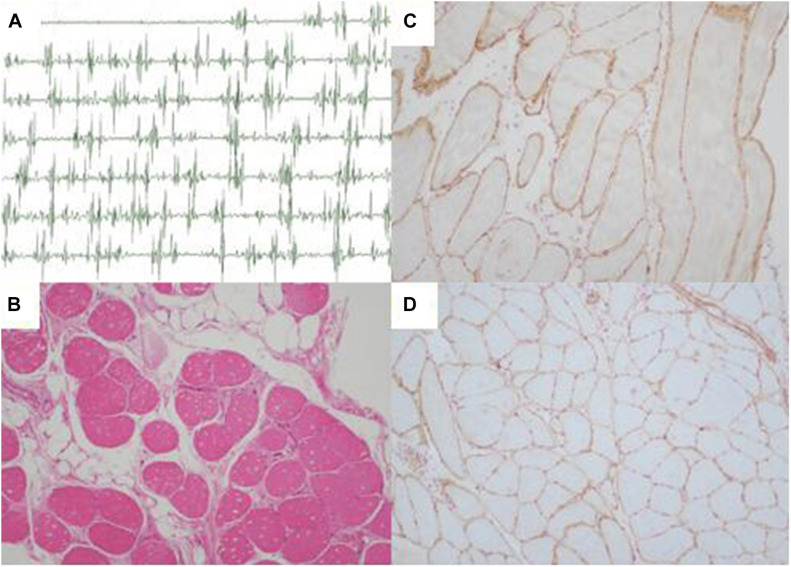
Electromyography and muscle biopsy morphologic findings of LGMD patient (II-3). **(A)** Electromyography showed myopathic features in the upper proximal muscles. **(B)** Regenerating fiber and a slight increase of connective tissue (H&E ×100). **(C)** Normal sarcolemmal expression of α-sarcoglycan (×100). **(D)** Normal sarcolemmal expression of β-sarcoglycan (×100). H&E, hematoxylin and eosin.

A three-generation Han Chinese pedigree was recruited at the Third Xiangya Hospital, Central South University, Changsha, Hunan, China (Figure 1A). The proband (II-3) is a 49-year-old female who was born to non-consanguineous parents. She presented a progressive proximal muscle weakness and initially had complaints of upper limb weakness and unable to run at age 39. At present, she had difficulty in raising arms, walking at a fast pace and getting up from squatting position. She had no complaints of distal muscle weakness, myalgia, dysphagia, dysarthria, ptosis, or numbness. Neurologic examinations revealed evident bilateral winging scapula, atrophy of both deltoids and shoulder girdle muscles, and pseudo-hypertrophy of bilateral upper arm muscles. Power testing revealed grade 4/5 at both proximal lower limbs and upper limbs, and 5/5 at both distal muscles. Sensory examination was normal. Her CK was elevated at 260 U/L (normal range 40–200 U/L). The electromyography (EMG) showed myopathic features in the upper proximal muscles (Figure 2A). She has no significant extramuscular symptom. The 46-year-old younger sister (II-5) suffered from similar weakness in limbs for ten years and had the same progressive history as the proband. The proband’s mother (I-2) is 81 years old, whose initial symptom is mild weakness of her arms and difficulty in running at the age of 41 years. The disorder was slowly progressive. At present, she is unable to walk and raise arms above the head (Table 1).

**TABLE 1:** Clinical characteristics of patients in the pedigree with LGMD.

Patients	Sex/age (years)	Age of onset (years)	Visible muscle atrophies	Specific features	Muscular strength	CK level (U/L)	Heart involvement
I-2	Female/81	41	Bilateral winging scapula, atrophy of both deltoids and shoulder girdle muscles	Proximal lower limb and upper limb muscles weakness, unable to walk and raise arms above the head	4/5 at both proximal lower limbs and upper limbs	N/A	–
II-3	Female/49	39	Evident bilateral winging scapula, atrophy of both deltoids and shoulder girdle muscles, and pseudo-hypertrophy of bilateral upper arm muscles	Proximal lower limb and upper limb muscles weakness, have difficulty in raising arms, walking at a fast pace and getting up from squatting position	4/5 at both proximal lower limbs and upper limbs	260	–
II-5	Female/46	36	Evident bilateral winging scapula, atrophy of both deltoids and shoulder girdle muscles, and pseudo-hypertrophy of bilateral upper arm muscles	Proximal lower limb and upper limb muscles weakness, have difficulty in raising arms, walking at a fast pace and getting up from squatting position	4/5 at both proximal lower limbs and upper limbs	N/A	–

**“CK” creatine kinase, “LGMD” limb-girdle muscular dystrophy, “N/A” not available, “–” without this symptom.**

Peripheral blood samples were drawn from nine pedigree members, including three affected individuals (I-2, II-3, and II-5) and six unaffected members (II-1, II-2, II-4, III-1, III-2, and III-3). Blood samples were taken from 200 unrelated ethnicity-matched individuals lacking diagnostic specificity or a family history of LGMD (male/female: 100/100, age 47.2 ± 6.4 years). Informed consent was obtained from all subjects, and this study was approved by the Institutional Review Board of the Third Xiangya Hospital, Central South University.

### Histopathology and Immunohistochemistry

Patient II-3′s biopsy samples of biceps brachii were obtained. The prepared cryosections were stained with hematoxylin and eosin (H&E). Frozen sections were performed immunohistochemically using a group of primary antibodies, including α-sarcoglycan (1:50), β-sarcoglycan (1:50), γ-sarcoglycan (1:50), and δ-sarcoglycan (1:25) (Charton et al., 2010; Liang et al., 2015). Primary and secondary antibodies were obtained from OriGene Technologies Inc. (Rockville, MD, United States) and Fuzhou Maixin Biotech Co., Ltd. (Fuzhou, China).

### Whole Exome Sequencing

Genomic DNA was extracted in accordance with a normative phenol-chloroform extraction method (Yuan et al., 2015). Whole exome sequencing for two patients (II-3 and II-5) was performed by BGI-Shenzhen (Shenzhen, China). The captured exome library was sequenced on Illumina HiSeq 2000 (Illumina, Inc., San Diego, CA, United States).

### Read Mapping and Variant Analysis

Burrows-Wheeler Aligner (BWA) software mapped clean reads to the reference human genome sequence. Alignment results were obtained after duplicate removal using Picard tools (v2.5.0)^1^, and base quality recalibration and local alignment by Genome Analysis Toolkit (v3.6). Single nucleotide polymorphisms (SNPs) and insertions-deletions (indels) were annotated using SnpEff software^2^. Variant filtrations were performed using data from the Single Nucleotide Polymorphism database (dbSNP) v141, 1000 Genomes Project, the NHLBI-Exome Sequencing Project (ESP) 6500, the Exome Aggregation Consortium (ExAC), and BGI in-house exome databases, and the variants with the frequency <1% were retained. Non-synonymous variants located in exonic regions and variants in canonical splicing sites were prioritized. Amino acid substitution affection for the protein structure or function was assessed by MutationTaster^3^ and Polymorphism Phenotyping version 2 (PolyPhen-2)^4^ for functional prediction (Yuan et al., 2015; Hu et al., 2017).

Locus-specific PCR primers for potential variants were designed as previously described (Yuan et al., 2015), and Sanger sequencing was performed for variant validation. Sequences of the primers were: (*TTN* variant) 5′-CCATCCTTA AACCACTGAGCA-3′ and 5′-GTTTTGCTGTTTGCATTGGA-3′, (*TRAPPC11* variant) 5′-AAGCCATAAGTGGGGAGCTA-3′ and 5′-ATCACTGGGCTCCACAGAAA-3′. Conservation analyses were performed using the Basic Local Alignment Search Tool (BLAST)^5^. The potential interactions of two genes were further analyzed using the GeneMANIA (v3.6.0)^6^ (Franz et al., 2018).

## Results

### Pathological Findings

Pathological findings of II-3′s muscle biopsy samples showed variation in fiber size, atrophic fibers, regenerating fibers, internally placed nuclei, and a slight increase of connective tissue (Figure 2B). Immunohistochemistry analysis of sarcolemmal proteins showed no apparent abnormality staining with α-, β-, γ- and δ-sarcoglycan (Figures 2C,D).

### Molecular Findings

We obtained 50,621,019 and 50,390,601 bp in target region in patients II-3 and II-5, respectively. Target regions mean sequencing depths were 105.88× and 152.47×. Target exome regions coverage was 99.54 and 99.64%, respectively. A total of 109,353 SNPs and 18,362 indels were identified in patient II-3. A total of 114,131 SNPs and 19,559 indels were identified in patient II-5. Scheme prioritization discriminated the likely pathogenic changes in patients, which was similar to recent genetic studies (Zheng et al., 2016b; Hu et al., 2017). After being filtered against public databases and BGI in-house exome databases, 54 heterozygous potential disease-causing variants were identified (Supplementary Table 1).

**FIGURE 3:**
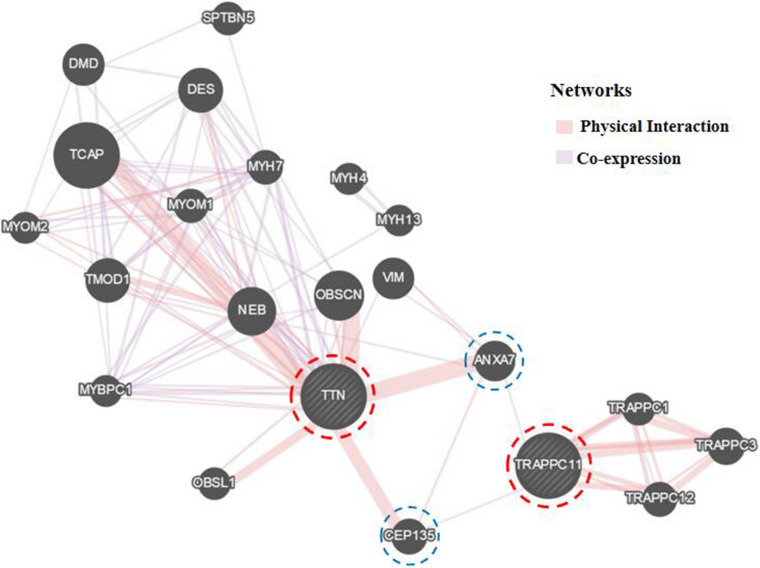
Biological interaction network of *TTN* and *TRAPPC11* (GeneMANIA v3.6.0, http://genemania.org). *TTN*, the titin gene; *TRAPPC11*, the trafficking protein particle complex 11 gene.

Further Sanger sequencing and co-segregation analysis of these variants in the pedigree excluded every heterozygous variant as a single genetic cause of autosomal dominant LGMD. Only the digenic variants *TTN* (NM_001267550.2) c.19481T>G (p.Leu6494Arg) and *TRAPPC11* (NM_021942.5) c.3092C>G (p.Pro1031Arg) were observed in all three patients (I-2, II-3, and II-5, Figures 1B,C), co-segregating with this family’s disorder phenotype, and those (II-1, II-2, III-1, and III-2) carrying either the *TTN* or the *TRAPPC11* variant were asymptomatic. Other unaffected family members (II-4 and III-3) and none of the 200 controls had either the *TTN* c.19481T>G variant or the *TRAPPC11* c.3092C>G variant (Figures 1D,E). The identified *TTN* c.19481T>G and *TRAPPC11* c.3092C>G variants have a low frequency recorded in the above public databases, including 1000 Genomes Project (4×10^–4^ and 3.4×10^–3^, respectively), the NHLBI-ESP6500 (8×10^–5^, both), and ExAC (9.9×10^–5^ and 9.6×10^–4^, respectively). Bioinformatics softwares predicted that two variants are “disease causing” (MutationTaster) and “possibly damaging” (PolyPhen-2), respectively. TTN protein p.Leu6494 and TRAPPC11 protein p.Pro1031 are highly conserved in different species. *TRAPPC11* is co-expressed with the annexin A7 gene (*ANXA7*) and the centrosomal protein 135 gene (*CEP135*) which both have physically interaction with *TTN* using GeneMANIA (Figure 3). These results suggest a possible disease-causing role of the two identified variants.

## Discussion

This study identified digenic variants, *TTN* c.19481T>G and *TRAPPC11* c.3092C>G, in a Han Chinese LGMD family. Three individuals (I-2, II-3, and II-5) carrying both variants presented proximal muscle weakness, after age 35, which is compatible with LGMD. Carriers in the family of either *TTN* or *TRAPPC11* variant, singly, were unaffected. Digenic variants (*TTN* c.19481T>G and *TRAPPC11* c.3092C>G) co-segregated with the LGMD phenotype in this pedigree. Our subjects had adult-onset age, slow progression and mildly elevated CK, different from either typical LGMD2J or LGMD2S (Nigro and Savarese, 2014; Zheng et al., 2016a).

The *TTN* gene (MIM 188840) has 363 exons and theoretically encodes a 3,960 kDa protein with 35,991 residues (Savarese et al., 2016). TTN, the largest human protein, spans half of sarcomere from the Z-disk to the M-line, extending longer than one micrometer (Chauveau et al., 2014; Gigli et al., 2016). TTN proteins form a third filament system in striated muscle (Dos Remedios and Gilmour, 2017; Kellermayer et al., 2019). The TTN protein is composed of four diverse domains: the amino-terminal Z-line, the I-band, A-band regions, and the carboxy-terminal M-line extremity (Neiva-Sousa et al., 2015; Gigli et al., 2016). The TTN p.Leu6494Arg variant is located on the I-band, which contains numerous protein partner-binding sites and multiple elastic spring elements with highly repetitive domains, associated with its elasticity (Chauveau et al., 2014; Neiva-Sousa et al., 2015). Mice with a shortened TTN elastic tandem immunoglobulin (Ig)-like segment showed significant spine curvature and muscle fiber atrophy (Buck et al., 2014).

The *TRAPPC11* gene (MIM 614138) encodes a 1,133-amino acid protein containing two functional regions (the foie gras and gryzun domains) (Matalonga et al., 2017). This protein is a subunit of the TRAPP III multiprotein complex. It is involved in anterograde transport from the endoplasmic reticulum (ER) to the ER-to-Golgi intermediate compartment (ERGIC) and proposed to participate in regulating N-linked glycosylation (Scrivens et al., 2011; Koehler et al., 2017; Matalonga et al., 2017). *TRAPPC11* knockdown in HeLa cells led to Golgi apparatus dispersal, defective protein glycosylation, and lipid accumulation (Wendler et al., 2010; Scrivens et al., 2011; DeRossi et al., 2016). The *trappc11*-mutant zebrafish larvae had motility defects (DeRossi et al., 2016).

To date, at least 54 human digenic diseases involved in 169 genes have been recorded in DIDA (DIgenic diseases DAtabase)^7^. In DIDA, five relationship types are defined for digenic combination: directly interaction, indirectly interaction, pathway membership, co-expression and similar function (Gazzo et al., 2016). *TTN* gene variants have previously been implicated with variants in other genes as the basis for familial hypertrophic cardiomyopathy, left-ventricular non-compaction cardiomyopathy, and dilated cardiomyopathy (Waldmüller et al., 2015). Double heterozygous mutations in two genes were identified in several patients with genetic myopathies (Trabelsi et al., 2008; Peddareddygari et al., 2018; Chakravorty et al., 2020). The bioinformatics analysis revealed possible interactions and common pathways in desmin and calpain 3, which supported the two heterozygous variants leading to the patient’s LGMD in a digenic mechanism (Peddareddygari et al., 2018). Our study suggests a possible causative role of digenic variants in *TTN* and *TRAPPC11* genes in the LGMD phenotype apparently segregating in a dominant way in the studied family.

Both TTN, an abundant sarcomeric multifunctional protein, and TRAPPC11, an intracellular vesicle trafficking protein in biosynthesis, are reported to be related to LGMD (Scrivens et al., 2011; Bögershausen et al., 2013; Neiva-Sousa et al., 2015). Intriguingly, the results of GeneMANIA demonstrated that *TTN* has physical interaction with *ANXA7* and *CEP135*, while *TRAPPC11* is co-expressed with *ANXA7* and *CEP135*. Those may imply an identical potential pathogenic pathway involving TTN and TRAPPC11 proteins resulting in the myopathy. The interrelationship and synergy between the two proteins await further functional experiments. Though with the application of appropriate approaches for diagnosis, some LGMD cases remain undiagnosed in genetics, and monogenic variants may not be accountable for all cases (Magri et al., 2017; Yu et al., 2017; Chakravorty et al., 2020). Although the possibility of complex rearrangement, deletion, and duplication involving one or a few exons, or deep pathogenic point variants in introns cannot be fully ruled out, our study provides the clue of a possible digenic mechanism or enhanced susceptibility responsible for the phenotype in this LGMD family.

## Conclusion

In summary, digenic variants *TTN* c.19481T>G (p.Leu6494Arg) and *TRAPPC11* c.3092C>G (p.Pro1031Arg) were observed in a LGMD family and co-segregated with the disease phenotype, which may be responsible for the LGMD phenotype. However, our study cannot exclude the missed inspection such as complex rearrangement, gross deletion and gross duplication, as well as deep pathogenic point variants in introns involved in monogenic LGMD, presenting autosomal dominant or pseudo-dominant phenomenon. Our study provides a possibility of a digenic mechanism in unsolved families with muscular dystrophies.

## Data Availability Statement

The raw data presented in this article will be available by the authors, and reasonable requests to access the datasets by the qualified researchers should be directed to the corresponding author after legal permission.

## Ethics Statement

The studies involving human participants were reviewed and approved by the Institutional Review Board of the Third Xiangya Hospital, Central South University. Written informed consent to participate in this study was provided by the participants or their legal guardian/next of kin.

## Author Contributions

QC, LY, and HD contributed to designing the study, performing experiments, interpreting data, discussing contents, and writing the article. WZ, HX, YY, and ZS contributed to providing clinical data, analyzing data, and performing experiments. All authors reviewed the manuscript.

## Conflict of Interest

The authors declare that the research was conducted in the absence of any commercial or financial relationships that could be construed as a potential conflict of interest.

## Footnotes

1 http://broadinstitute.github.io/picard/

2 http://snpeff.sourceforge.net/SnpEff_manual.html

3 http://www.mutationtaster.org/

4 http://genetics.bwh.harvard.edu/pph2/

5 http://www.ncbi.nlm.nih.gov/BLAST/

6 http://genemania.org/

7 http://dida.ibsquare.be/browse/
