# Corrigendum: Digenic Variants in the *TTN* and *TRAPPC11* Genes Co-segregating With a Limb-Girdle Muscular Dystrophy in a Han Chinese Family

**DOI:** 10.3389/fnins.2021.678618

**Published:** 2021-04-21

**Authors:** Qian Chen, Wen Zheng, Hongbo Xu, Yan Yang, Zhi Song, Lamei Yuan, Hao Deng

**Affiliations:** ^1^Center for Experimental Medicine, The Third Xiangya Hospital, Central South University, Changsha, China; ^2^Department of Pathology, The Third Xiangya Hospital, Central South University, Changsha, China; ^3^Department of Neurology, The Third Xiangya Hospital, Central South University, Changsha, China; ^4^Disease Genome Research Center, Central South University, Changsha, China

**Keywords:** limb-girdle muscular dystrophies, digenic variants, the *TTN* gene, the *TRAPPC11* gene, exome sequencing

In the original article, there was an error in *Conclusion* as published.

“*TTN* c.3092C greater than G (p.Leu6494Arg)” should be changed to “*TTN* c.19481T greater than G (p.Leu6494Arg)”. The corrected section appears below.

In summary, digenic variants *TTN* c.19481T greater than G (p.Leu6494Arg) and *TRAPPC11* c.3092C greater than G (p.Pro1031Arg) were observed in a LGMD family and co-segregated with the disease phenotype, which may be responsible for the LGMD phenotype. However, our study cannot exclude the missed inspection such as complex rearrangement, gross deletion and gross duplication, as well as deep pathogenic point variants in introns involved in monogenic LGMD, presenting autosomal dominant or pseudo-dominant phenomenon. Our study provides a possibility of a digenic mechanism in unsolved families with muscular dystrophies.

In addition, there was a mistake in the caption for Figure 1 as published. Within the description for Figure 1B, “*TTN* c.3092C greater than G (p.Leu6494Arg)” should be changed to “*TTN* c.19481T greater than G (p.Leu6494Arg)”. The corrected caption appears below.

(Figure 1). Pedigree and genetic data of the individuals in this study. **(A)** Pedigree of a Han Chinese three-generation family with LGMD. Arrow symbolizes proband; N, normal allele; V_1_, *TTN* c.19481T greater than G variant; V_2_, *TRAPPC11* c.3092C greater than G variant. **(B)** The sequencing diagram of heterozygous *TTN* c.19481T greater than G (p.Leu6494Arg) variant. **(C)** The sequencing diagram of heterozygous *TRAPPC11* c.3092C greater than G (p.Pro1031Arg) variant. **(D)** Sequence of normal control in the *TTN* gene. **(E)** Sequence of normal control in the *TRAPPC11* gene. LGMD, limb-girdle muscular dystrophies; *TTN*, the titin gene; *TRAPPC11*, the trafficking protein particle complex 11 gene.

The authors apologize for these errors and state that they do not change the scientific conclusions of the article in any way. The original article has been updated.

