# Supplementary material for: Digenic Variants in the TTN and TRAPPC11 Genes Co-segregating With a Limb-Girdle Muscular Dystrophy in a Han Chinese Family
Source: Front Neurosci. 2021 Mar 4;15:601757. doi: 10.3389/fnins.2021.601757 (PMC7969792; doi:10.3389/fnins.2021.601757)
Supplement: Supplementary file 1 [file Table_1.pdf]

Supplementary Material

Supplementary Table 1 The potential disease-causing gene variants identified in patients (II-3 and II-5) by whole exome sequencing

No.	Chromosome/position	Gene symbol*	Reference sequence	Variant
1	Chr 1/3413552	*MEGF6*	NM_001409.3	c.3613C>T, p.R1205*
2	Chr 1/3703514	*LRRC47*	NM_020710.3	c.976A>G, p.S326G
3	Chr 1/23966181	*MDS2*	NM_001348075.2	c.158T>G, p.L53R
4	Chr 1/186359880	*ODR4*	NM_017847.5	c.512G>T, p.G171V
5	Chr 1/206661284	*IKBKE*	XM_005273356.1	c.1650G>C, p.M550I
6	Chr 1/247464533	*ZNF496*	XM_005273328.1	c.1160T>G, p.V387G
7	Chr 2/17997931	*MSGN1*	NM_001105569.3	c.146C>T, p.P49L
8	Chr 2/112843588	*TMEM87B*	NM_032824.2	c.845G>A, p.G282D
9	Chr 2/179593070	*TTN*	NM_001267550.2	c.19481T>G, p.L6494R
10	Chr 2/201369550	*KCTD18*	NM_152387.2	c.293C>A, p.P98H
11	Chr 3/120973797	*STXBP5L*	NM_014980.2	c.1497G>T, p.K499N
12	Chr 3/160156169	*TRIM59*	XM_005247391.1	c.887C>T, p.P296L
13	Chr 4/48552688	*FRYL*	XM_005248082.1	c.4875C>G, p.N1625K
14	Chr 4/120213519	*USP53*	NM_019050.2	c.2375A>G, p.K792R
15	Chr 4/184627996	*TRAPPC11*	NM_021942.5	c.3092C>G, p.P1031R
16	Chr 4/187074881	*FAM149A*	NM_015398.4	c.169_170insGACCCCC, p.L60fs
17	Chr 5/1037638	*NKD2*	NM_001271082.1	c.791C>T, p.S264F
18	Chr 5/52201707	*ITGA1*	NM_181501.1	c.1424T>A, p.I475N
19	Chr 5/109178158	*MAN2A1*	NM_002372.4	c.2696A>G, p.Y899C
20	Chr 7/1510571	*INTS1*	NM_001080453.2	c.6368C>G, p.P2123R
21	Chr 7/97822697	*LMTK2*	NM_014916.4	c.2920A>G, p.S974G
22	Chr 7/122338841	*RNF133*	NM_139175.1	c.132A>G, p.I44M
23	Chr 7/150935727	*CHPF2*	XM_005250015.1	c.2096C>T, p.A699V
24	Chr 7/151078656	*WDR86*	XM_005249988.1	c.1207C>T, p.P403S
25	Chr 8/90926850	*OSGIN2*	NM_001126111.1	c.404C>G, p.T135R
26	Chr 9/134397577	*POMT1*	NM_007171.3	c.2035G>A, p.V679M
27	Chr 9/138418315	*LCN1*	NM_001252618.1	c.655delC, p.A221fs
28	Chr 10/46999656	*GPRIN2*	XM_005270332.1	c.776C>T, p.P259L

No.	Chromosome/position	Gene symbol*	Reference sequence	Variant
29	Chr 10/50013389	*WDFY4*	XM_005270005.1	c.4569C>G, p.I1523M
30	Chr 10/102766630	*LZTS2*	NM_032429.4	c.1715G>C, p.R572P
31	Chr 10/135096593	*TUBGCP2*	NM_001256617.1	c.2362A>G, p.N788D
32	Chr 11/22281251	*ANO5*	XM_005252822.1	c.1516T>C, p.F506L
33	Chr 11/62554434	*TAF6L*	NM_006473.3	c.1535C>A, p.S512*
34	Chr 12/319017	*SLC6A12*	NM_001122848.2	c.136G>A, p.V46M
35	Chr 12/6127726	*VWF*	NM_000552.5	c.4858C>T, p.P1620S
36	Chr 12/48062768	*RPAP3*	NM_024604.3	c.1644C>G, p.N548K
37	Chr 12/50386143	*RACGAP1*	XM_005268811.1	c.1520A>T, p.H507L
38	Chr 12/55795106	*OR6C65*	NM_001005518.1	c.794G>T, p.G265V
39	Chr 12/106532238	*NUAK1*	NM_014840.2	c.194C>A, p.T65N
40	Chr 12/120534220	*RAB35*	XM_005253826.1	c.816_829del, p.M272fs
41	Chr 14/45579368	*PRPF39*	NM_017922.3	c.1248T>A, p.H416Q
42	Chr 14/64690070	*SYNE2*	NM_182914.3	c.20354A>C, p.Q6785P
43	Chr 14/89016702	*PTPN21*	NM_007039.3	c.60G>T, p.K20N
44	Chr 15/62211567	*VPS13C*	NM_020821.2	c.7559C>G, p.A2520G
45	Chr16/1604819	*TMEM204*	NM_001256541.1	c.473G>A, p.R158K
46	Chr 16/2293361	*ECI1*	NM_001919.4	c.521T>C, p.I174T
47	Chr 16/67006304	*CES3*	NM_024922.5	c.1337G>A, p.S446N
48	Chr 17/12847454	*ARHGAP44*	NM_014859.4	c.802A>G, p.I268V
49	Chr 17/30648273	*RHBDL3*	XM_005257935.1	c.1139G>A, p.R380K
50	Chr 17/35343961	*AATF*	ENST00000225402.5	c.878A>G, p.Q293R
51	Ch r17/73127637	*NT5C*	NM_001252377.1	c.166G>C, p.D56H
52	Chr 18/2760723	*SMCHD1*	NM_015295.2	c.4420C>T, p.L1474F
53	Chr 19/40421117	*FCGBP*	NM_003890.2	c.2804G>C, p.R935P
54	Chr 19/56733965	*ZSCAN5A*	XM_005259254.1	c.893G>A, p.S298N

*Gene symbol is approved by HGNC (HUGO Gene Nomenclature Committee).
